# Cerebrospinal fluid ctDNA clarifies clonal divergence: leptomeningeal flare of *EGFR*-mutant disease after switch to selpercatinib for acquired *RET* fusion

**DOI:** 10.1016/j.jlb.2025.100450

**Published:** 2025-12-02

**Authors:** Kei Kunimasa, Motohiro Tamiya, Takako Inoue, Nobuaki Mamesaya, Tsunehiro Tanaka, Kiyohide Komuta, Shun Futamura, Keiichiro Honma, Kazumi Nishino

**Affiliations:** a Department of Thoracic Oncology, Osaka International Cancer Institute, Osaka, Japan; b Department of Diagnostic Pathology and Cytology, Osaka International Cancer Institute, Osaka, Japan

**Keywords:** Cerebrospinal fluid, Circulating tumor DNA, Leptomeningeal metastasis, *RET* fusion, Clonal divergence, *EGFR* exon 19 deletion

## Abstract

Liquid biopsy can expose spatially segregated resistance biology that is invisible to single-site tissue testing, particularly across the blood–brain barrier. We report how cerebrospinal fluid (CSF) circulating tumor DNA (ctDNA) clarified therapeutic direction in *EGFR*-mutated non-small-cell lung cancer (NSCLC) with leptomeningeal involvement. A 67-year-old woman with *EGFR* exon 19–deleted adenocarcinoma received afatinib followed by long-term osimertinib. After three years, progression of the primary lesion prompted rebiopsy, which revealed a *CCDC6-RET* fusion with strong RET immunoreactivity. Selpercatinib monotherapy yielded minor thoracic shrinkage at 1 month but was followed by dizziness and MRI evidence of leptomeningeal enhancement at 3 months. CSF analysis showed pleocytosis without malignant cells. Critically, CSF ctDNA demonstrated the *EGFR* E746_A750 deletion at 29.2 % variant allele frequency by amplicon sequencing, whereas the *CCDC6–RET* fusion was undetectable by targeted sequencing and highly sensitive single-plex qPCR using junction-specific primers. Re-challenging with osimertinib rapidly improved symptoms and led to resolution of leptomeningeal enhancement. These data indicate clonal divergence at acquired resistance: a *RET*-fusion clone dominated the thoracic compartment while *EGFR*-addicted clones predominated in the leptomeninges. The leptomeningeal flare after discontinuing EGFR inhibition highlights the risk of switching to RET inhibitor monotherapy when CNS disease is driven by the original *EGFR* mutant clone. CSF liquid biopsy provided actionable, compartment-specific genotyping that outperformed cytology and guided effective retreatment. Incorporating CSF ctDNA into routine evaluation may improve therapeutic alignment across sanctuary sites; when feasible, maintaining EGFR blockade should be considered when CNS involvement is suspected in *EGFR*-mutated NSCLC in routine practice.

## Highlights

• Novel targetable driver alterations may appear during EGFR-TKI resistance.
• We present a case in which *CCDC6*-*RET* fusion was detected as a resistance mutation.
• Switching to selpercatinib monotherapy led to rapid onset of cancerous meningitis.
• Re-switching to osimertinib monotherapy resulted in rapid improvement of meningitis.
• NGS analysis using cell free DNA from spinal fluid showed a tumor heterogeneity.

## 1 Introduction

Osimertinib, a third-generation irreversible oral epidermal growth factor receptor-tyrosine kinase inhibitor (EGFR-TKI) [1], was developed as a selective inhibitor of both EGFR-TKI-sensitizing and resistant *EGFR* T790M mutations [2,3]. Clinical trials have demonstrated the efficacy of osimertinib against brain metastases in patients with *EGFR*-mutated non-small cell lung cancer (NSCLC) who have experienced disease progression with first- and second-generation EGFR-TKIs [4,5]. Although initially developed to overcome the *EGFR* T790M resistant mutation, osimertinib is now used as a primary treatment because of its superior blood-brain barrier penetration, as evidenced by the results of the phase III FLAURA trial [3], which compared the effects of first-line osimertinib with those of other EGFR-TKIs in patients with *EGFR*-mutated NSCLC. Consequently, osimertinib has become the mainstay first-line treatment for patients with *EGFR*-mutated NSCLC, and overcoming this resistance remains a significant clinical challenge.

The mechanisms of acquired resistance to EGFR-TKIs in *EGFR*-mutated NSCLC are diverse, including genomic mechanisms such as point mutations, amplifications, and oncogenic fusions [6,7]. Resistance mechanisms to osimertinib can be broadly divided into EGFR-dependent and EGFR-independent [6,7]. The most frequent EGFR-dependent mechanism, reported to occur in 7–10 % of cases, is the C797S mutation at the ATP-binding site to which osimertinib binds [8]. Other *EGFR* mutations associated with resistance include those at G724 and L718 [9,10]. The most frequent EGFR-independent mechanism is MET (mesenchymal–epithelial transition factor) amplification, reported to occur in 7–15 % of cases [6,7]. However, other targetable driver alterations have been detected, albeit rarely. Additional targetable driver mutations, such as *BRAF* (B-Raf proto-oncogene, serine/threonine kinase) V600E, *RET* (rearranged during transfection proto-oncogene), *NTRK* (neurotrophic tyrosine receptor kinase), and *ROS1* (ROS proto-oncogene 1, receptor tyrosine kinase) fusions, have been identified [6,7,11]. Combination therapies with a molecularly targeted agent corresponding to resistant alterations with EGFR-TKIs are expected to overcome resistance. However, these combination therapies are still undergoing clinical trials and are challenging to apply in clinical practice.

We present a case in which a *CCDC6 (*coiled-coil domain containing 6)-*RET* fusion (*CCDC6* [1]*RET* [11] fusion) was detected as an osimertinib-resistant mutation, and a patient was switched from osimertinib to selpercatinib monotherapy, resulting in a rapid onset of carcinomatous meningitis. Analysis using cell-free DNA from the cerebrospinal fluid suggested that *EGFR*-mutant clones were the primary cause of carcinomatous meningitis.

## 2 Case presentation

**Fig. 1:**
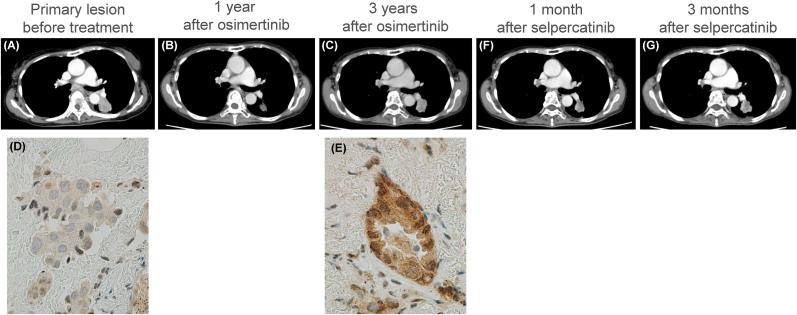
Serial images of enhanced chest computed tomography. (A) Before treatment, (B) 1 year after osimertinib treatment, (C) 3 years after osimertinib treatment, (D) 1 month after selpercatinib treatment, (E) 3 months after selpercatinib treatment. Immunostaining images of bronchoscopic biopsy specimens of primary lung lesion with anti-RET antibody (#ab134100). (F) Before osimertinib treatment and (G) at osimertinib resistance.

**Fig. 2:**
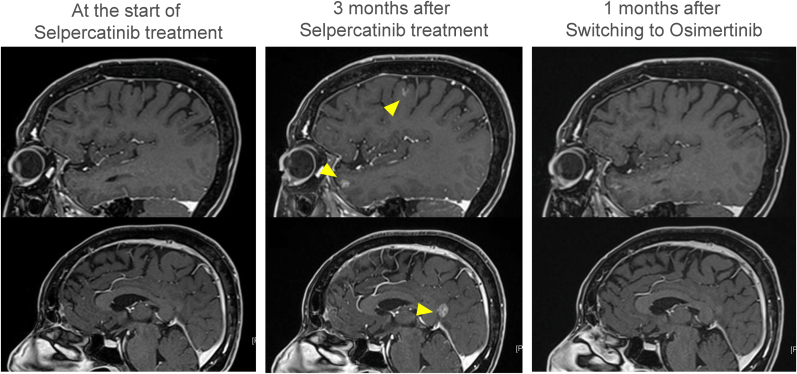
Sagittal images of brain enhanced magnetic resonance imaging. No enhanced lesion was observed at the start of selpercatinib treatment, but 3 months after starting selpercatinib, lesions with contrast effects appeared (yellow arrowheads) and enhancement images of the cerebral sulci were observed. One month after switching to osimertinib treatment, these contrast effects disappeared.

A 67-year-old woman with back pain and lower-extremity weakness presented to our hospital. Computed tomography revealed a 25-mm mass in the left lower lobe (Fig. 1A) and multiple masses in the thoracic vertebrae. Histopathologic analysis of the lung mass biopsy specimen revealed adenocarcinoma. Genetic profiling of the tumor revealed an *EGFR* exon 19 p. E746_A750del mutation; therefore, she was treated with afatinib as first-line chemotherapy. After approximately 1 month of afatinib treatment, the patient developed severe diarrhea and was switched to osimertinib due to intolerable toxicities. Switching to osimertinib improved the diarrhea, and treatment with osimertinib was continued with mild adverse skin events (Fig. 1B). After approximately 3 years of treatment, the primary left lower lobe lesion gradually increased in size (Fig. 1C) and was re-biopsied. Genetic analysis of the specimen revealed a *CCDC6*-*RET* fusion (CCDC6-intron1-RET-intron11; chr10:61639130-chr10:43611591 (hg19 genome)) in addition to the *EGFR* exon 19 p. E746_A750del. No fusion was detected in the specimens collected before EGFR-TKI administration. When the patient was switched from osimertinib to selpercatinib monotherapy, the primary lesion slightly reduced 1 month after treatment (Fig. 1D) but tended to increase again 3 months later (Fig. 1E), accompanied by severe light-headedness and dizziness. Immunostaining using an anti-RET antibody (#ab134100) showed strong staining only in the rebiopsied sample (Fig. 1F and G). Brain-enhanced magnetic resonance imaging (MRI) revealed new metastatic brain lesions suggestive of carcinomatous meningitis. Cerebrospinal fluid examination revealed an elevated number of cells; however, no malignant cells were detected. When the treatment was switched back to osimertinib, the patient's clinical symptoms improved rapidly, and head contrast-enhanced MRI showed a trend toward resolution of the brain metastases (Fig. 2).

**Fig. 3:**
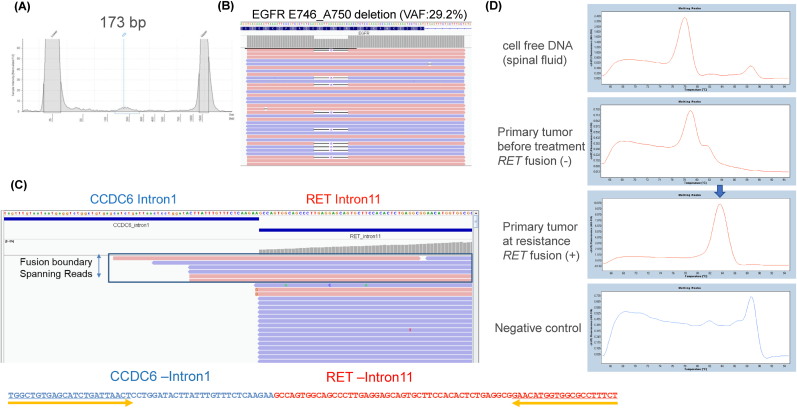
(A) Cell-free DNA with a peak around 173 bp was extracted from the spinal fluid. (B) The EGFR E746_A750 deletion allele was detected at 29.2 % frequency by amplicon target sequence using cell-free DNA. (C) Integrative genomics viewer of the CCDC6-RET fusion and primer design of boundary region of the fusion. (D) Result of quantitative RT-PCR for the boundary region. qRT-PCR showed peaks in lung tissue sample at the time of osimertinib resistance (positive control) but not in cell-free DNA from the spinal fluid and lung tumor before treatment. **Extraction of cell free DNA from spinal fluid**: The cfDNA fraction from cerebrospinal fluid specimen was purified using the QIAamp Circulating Nucleic Acid Kit (Qiagen). For the FFPE (formalin-fixed, paraffin-embedded) specimens of diagnostics point and of progressive disease (PD) point, DNA and RNA were purified using the Maxwell® RSC DNA FFPE Kit and Maxwell® RSC RNA FFPE Kit (Promega, WI, USA) according to the manufacturer's protocol. The QubitTM fluorometer (Thermo Fisher, USA) with dsDNA HS (High Sensitivity) Assay Kits was used for the quantification of genomic DNA. **Amplicon sequencing for EGFR Ex.19 deletion mutation:** Mutation analysis of EGFR was performed by DNA module of compact panel assay [20]. For mutation analysis of driver mutations, multiplex PCR using KOD-Plus-Neo (Toyobo, Osaka, Japan) was performed to amplify EGFR (exon 18–21). Forty cycles of 98 °C for 10 s and 62 °C for 30 s were performed to amplify regions on the panel of DNA module. After purification with AMPure XP (Beckman Coulter Life Sciences), illumine sequence libraries were prepared from these PCR products using the GenNext® NGS Library Prep Kit (Toyobo). All steps were performed according to the manufacturer's protocols. The constructed sequence libraries were sequenced using MiSeq (Illumina, CA, USA) by paired-end mode (2 x 150 bp). **Sequencing for RET fusion and quantitative RT-PCR:** To reveal the CCDC6-RET fusion boundary breakpoint on the intron 11 of RET, tiling anchored PCR-NGS assay (tiling NOIR-SS) [21] was performed according to our previous protocol. PCR primers on the RET intron 11 were designed to cover entire region of RET intron 11 with 150–200 intervals [22]. 10 ng input of dsDNA was used for the assay. After detecting the CCDC6-RET fusion breakpoint on the RET intron11 and CCDC6 exon1, singleplex qPCR primer to detect fusion breakpoint was re-designed using Primer-BLAST. Singleplex qPCR assay was performed using KOD SYBR® qPCR Mix (Toyobo) according to the manufactures protocol. Thermal cycler condition was set as follows; 40 cycles of 98°10sec, 62°10sec, and 68°20sec.

To study the clonality of tumor cells in central lesions, cell-free DNA was extracted from the spinal fluid (Fig. 3A), and amplicon target sequencing was performed on *EGFR* Ex.19 deletions, which revealed an E746_A750 deletion at a variant allele frequency of 29.2 % (Fig. 3B). The same cell-free DNA was used to search for the *CCDC6*-*RET* fusion but was not detected. To increase detection sensitivity, singleplex quantitative polymerase chain reaction (qPCR) was performed on the fusion gene using PCR primers specific for the border region between the *CCDC6* and *RET* genes (Fig. 3C); however, no amplification was observed in cell-free DNA from the spinal fluid. The same experiment was performed using a resistant lung tumor specimen as a positive control, and amplification of the boundary region was observed (Fig. 3D).

## 3 Discussion

Osimertinib is currently one of the most effective drugs available against *EGFR* major activating mutations, such as Ex.19 del and L858R, and overcoming this resistance is a critical issue. Drug concentration in the central nervous system (CNS) is lower than in the bloodstream due to the blood-brain barrier, making it harder to control and more prone to recurrence than in the rest of the body [12,13]. In the present case, when the treatment was switched to selpercatinib monotherapy for *RET* fusion, the patient developed a disease flare due to carcinomatous meningitis. Analysis of cell-free DNA from the spinal fluid implied that the primary seeding lesions of the meninges could have been *EGFR* exon 19 p. E746_A750del clones. Although direct examination of meningeal dissemination was not feasible, the appearance of *CCDC6* [1]*RET* [11] fusion may be a localized phenomenon in the resistant area of the primary pulmonary lesion.

Despite their low incidence, oncogenic fusion genes have also been implicated in osimertinib resistance mechanisms [11], including *CCDC6* [1]*RET* [11] fusion. In both mouse models and clinical case reports, additional targetable driver alterations were often ineffective when treated with the corresponding molecularly targeted agents as switching monotherapies [14]. Therefore, combination therapy with EGFR-TKIs has been demonstrated to be more effective [14]. A biomarker-directed phase II platform study is currently underway to assess the efficacy of combinational molecular targeted therapies based on osimertinib resistance mechanisms; the results should lead to the establishment of new molecular targeted therapies after osimertinib resistance acquisition [15]. There is also a recent report confirming the efficacy and safety of the combination of selpercatinib and osimertinib in the treatment of acquired resistant *RET* fusion [16]. Currently, combination therapy with osimertinib and molecularly targeted agents based on resistance mechanisms is not feasible in practice, and only switching to molecularly targeted agents based on resistance mechanisms, as in this case, can be attempted.

Selpercatinib is a potent and selective RET inhibitor with high CNS penetration and high control of brain metastases [17,18]. The patient developed cancerous meningitis 3 months after starting selpercatinib treatment, although no obvious brain metastases were detected on head-contrast MRI at the start of selpercatinib treatment. After discontinuing osimertinib and switching to selpercatinib, rapid onset of cancerous meningitis was observed, and the lack of amplification of the *CCDC6* [1]*RET* [11] fusion boundary by qRT-PCR (quantitative reverse-transcription polymerase chain reaction) using spinal fluid-derived cell-free DNA suggested that the brain metastases were likely predominant *EGFR* exon 19 p. E746_A750del mutated clones. The clinical course seems to support this hypothesis, as osimertinib rechallenge rapidly improved the cancerous meningitis. Administration of molecularly targeted agents may increase heterogeneity within the tumor, forming a branched evolutionary pattern due to selective pressure [19]. In the present case, the *CCDC6* [1]*RET* [11] fusion clone might have developed osimertinib resistance only in the primary tumor.

This study has several limitations. A more detailed analysis of the clonality of tumor cells in central lesions would have been possible if tissue samples from the metastatic brain had been examined. However, the patient's load in clinical practice renders biopsies of central lesions impracticable owing to the high encumbrance of the patient. For the examination of cell-free DNA from the spinal fluid, cell-free RNA would have been more desirable for scrutinizing fusion genes. However, in the present study, the quantity of spinal fluid collected was insufficient and analysis with cell-free RNA was unattainable.

In the present study, we detected the *CCDC6* [1]*RET* [11] fusion as an osimertinib resistance mutation and switched the patient to selpercatinib monotherapy. However, the patient developed an acute exacerbation of cancerous meningitis, and analysis using cell-free DNA from the spinal fluid suggested meningeal dissemination mainly caused by *EGFR* exon 19 p. E746_A750del mutant clones, which might have contributed to the worsening of the cancerous meningitis on selpercatinib treatment. In the future, combination therapy with molecularly targeted agents and EGFR-TKIs may be desirable for additional targetable driver alterations after EGFR-TKI therapy.

## Availability of data and materials

The data that support the findings of this study are not publicly available because they contain information that could compromise the privacy of the research participants, but are available from the corresponding author (Kei Kunimasa, kei.kunimasa@oici.jp) upon reasonable request. Further inquiries can be directed to the corresponding authors.

## CRediT authorship contribution statement

Kei Kunimasa: Conceptualization, Formal analysis, Funding acquisition, Investigation, Methodology, Resources, Writing – original draft. Shun Futamura: Formal analysis, Investigation, Resources. Aki Kubota: Data curation, Formal analysis, Investigation, Methodology, Software and Writing – original draft. Kiyohide Komuta: Formal analysis, Investigation, Resources. Tsunehiro Tanaka: Formal analysis, Investigation, Resources. Takahisa Kawamura: Formal analysis, Investigation, Resources. Nobuaki Mamesaya: Formal analysis, Investigation, Resources. Takako Inoue: Formal analysis, Investigation, Resources. Motohiro Tamiya: Formal analysis, Investigation, Resources. Kazumi Nishino: Formal analysis, Investigation, Resources, Writing – review & editing.

## Ethical approval

The present study was approved by the ethics committee in our institute (#20136–3 and #22129). In addition, written informed consent was obtained from the patient for participation in this study and for publication of anonymized clinical information, images, and genetic findings.

## Declaration of generative AI in scientific writing

During the preparation of this manuscript, the authors used ChatGPT (OpenAI) solely to improve the readability and English phrasing of the text (e.g., polishing sentences and figure legends). ChatGPT was not used to generate, analyze, or interpret any data or scientific content. All AI-assisted text was reviewed and edited by the authors, who take full responsibility for the content of the published article.

## Funding sources

This work was supported by the 10.13039/501100001691Japan Society for the Promotion of Science (10.13039/501100001691JSPS) 10.13039/501100001691KAKENHI (Grant Number 22K16208).

## Declaration of competing interest

The authors declare the following financial interests/personal relationships which may be considered as potential competing interests: Kei Kunimasa reports financial support was provided by Japan Society for the Promotion of Science. Kei Kunimasa reports a relationship with Amgen Inc that includes: speaking and lecture fees. Kei Kunimasa reports a relationship with AstraZeneca Pharmaceuticals LP that includes: speaking and lecture fees. Kei Kunimasa reports a relationship with DAIICHI SANKYO COMPANY, LIMITED that includes: speaking and lecture fees. Kei Kunimasa reports a relationship with Eli Lilly Japan KK that includes: speaking and lecture fees. Kei Kunimasa reports a relationship with Janssen Pharmaceutical KK that includes: speaking and lecture fees. Kei Kunimasa reports a relationship with Merck & Co Inc that includes: speaking and lecture fees. Kei Kunimasa reports a relationship with MSD that includes: speaking and lecture fees. Kei Kunimasa reports a relationship with Novartis Pharmaceuticals Corporation that includes: speaking and lecture fees. Kei Kunimasa reports a relationship with Ono Pharmaceutical Co Ltd that includes: speaking and lecture fees. Kei Kunimasa reports a relationship with Pfizer Inc that includes: speaking and lecture fees. Kei Kunimasa reports a relationship with Chugai Pharmaceutical Co Ltd that includes: speaking and lecture fees. Motohiro Tamiya reports a relationship with Pfizer Inc that includes: speaking and lecture fees. Motohiro Tamiya reports a relationship with MSD that includes: speaking and lecture fees. Motohiro Tamiya reports a relationship with Chugai Pharmaceutical Co Ltd that includes: speaking and lecture fees. Motohiro Tamiya reports a relationship with Bristol Myers Squibb that includes: speaking and lecture fees. Motohiro Tamiya reports a relationship with Johnson and Johnson KK that includes: speaking and lecture fees. Motohiro Tamiya reports a relationship with Eli Lilly that includes: speaking and lecture fees. Motohiro Tamiya reports a relationship with AstraZeneca that includes: speaking and lecture fees. Motohiro Tamiya reports a relationship with Ono Pharmaceutical Co Ltd that includes: speaking and lecture fees. Motohiro Tamiya reports a relationship with Boehringer Ingelheim Ltd that includes: speaking and lecture fees. Motohiro Tamiya reports a relationship with Takeda Pharmaceutical Company Limited that includes: speaking and lecture fees. Takako Inoue reports a relationship with AstraZeneca that includes: speaking and lecture fees. Takako Inoue reports a relationship with Chugai Pharmaceutical Co Ltd that includes: speaking and lecture fees. Takako Inoue reports a relationship with Bristol Myers Squibb that includes: speaking and lecture fees. Takako Inoue reports a relationship with Ono Pharmaceutical Co Ltd that includes: speaking and lecture fees. Takako Inoue reports a relationship with MSD that includes: speaking and lecture fees. Kazumi Nishino reports a relationship with Ono Pharmaceutical Co Ltd that includes: funding grants. Kazumi Nishino reports a relationship with Taiho Pharmaceutical Co Ltd that includes: funding grants and paid expert testimony. Kazumi Nishino reports a relationship with Eli Lilly Japan KK that includes: funding grants. Kazumi Nishino reports a relationship with AbbVie Inc that includes: funding grants. Kazumi Nishino reports a relationship with DAIICHI SANKYO COMPANY, LIMITED that includes: funding grants. Kazumi Nishino reports a relationship with Amgen Inc that includes: funding grants and speaking and lecture fees. Kazumi Nishino reports a relationship with Eisai that includes: funding grants. Kazumi Nishino reports a relationship with Sanofi that includes: funding grants. Kazumi Nishino reports a relationship with Janssen Pharmaceuticals Inc that includes: funding grants and speaking and lecture fees. Kazumi Nishino reports a relationship with Novartis that includes: funding grants. Kazumi Nishino reports a relationship with Pfizer that includes: funding grants. Kazumi Nishino reports a relationship with Merck & Co Inc that includes: funding grants. Kazumi Nishino reports a relationship with Takeda Pharmaceutical Company Limited that includes: funding grants. Kazumi Nishino reports a relationship with AstraZeneca that includes: funding grants and speaking and lecture fees. Kazumi Nishino reports a relationship with Merus NV that includes: funding grants. Kazumi Nishino reports a relationship with MSD that includes: funding grants and speaking and lecture fees. Kazumi Nishino reports a relationship with Bayer Corporation that includes: funding grants. Kazumi Nishino reports a relationship with Delta-Fly Pharma, Inc. That includes: funding grants. Kazumi Nishino reports a relationship with IQVIA Holdings Inc that includes: funding grants. Kazumi Nishino reports a relationship with Nippon Boehringer Ingelheim Co Ltd that includes: funding grants. Kazumi Nishino reports a relationship with Parexel International (MA) Corporation that includes: funding grants. Kazumi Nishino reports a relationship with Asahi Kasei Pharma Corporation that includes: speaking and lecture fees. Kazumi Nishino reports a relationship with DAIICHI SANKYO COMPANY, LIMITED that includes: speaking and lecture fees. Kazumi Nishino reports a relationship with Eli Lilly Japan KK that includes: speaking and lecture fees. Kazumi Nishino reports a relationship with Kyorin Pharmaceutical Co Ltd that includes: speaking and lecture fees. Kazumi Nishino reports a relationship with Kyowa Kirin Co., Ltd that includes: speaking and lecture fees. Kazumi Nishino reports a relationship with Merck Biopharma Co., Ltd. That includes: speaking and lecture fees. Kazumi Nishino reports a relationship with Mochida Pharmaceutical Co Ltd that includes: speaking and lecture fees. Kazumi Nishino reports a relationship with Nippon Boehringer Ingelheim Co Ltd that includes: speaking and lecture fees. Kazumi Nishino reports a relationship with Nippon Kayaku Co Ltd that includes: speaking and lecture fees. If there are other authors, they declare that they have no known competing financial interests or personal relationships that could have appeared to influence the work reported in this paper.
